# Does HbA1c Level Have Clinical Implications in Diabetic Patients Undergoing Coronary Artery Bypass Grafting? A Systematic Review and Meta-Analysis

**DOI:** 10.1155/2017/1537213

**Published:** 2017-09-17

**Authors:** Jia Zheng, Jing Cheng, Tong Wang, Qian Zhang, Xinhua Xiao

**Affiliations:** ^1^Department of Endocrinology, Key Laboratory of Endocrinology, Ministry of Health, Peking Union Medical College Hospital, Diabetes Research Center of Chinese Academy of Medical Sciences & Peking Union Medical College, Beijing, China; ^2^Department of Cardiology, The Key Laboratory of Cardiovascular Remodeling and Function Research, Chinese Ministry of Education and Chinese Ministry of Public Health, Qilu Hospital of Shandong University, Jinan, China

## Abstract

**Aims/Introduction:**

The aim of the present study was to investigate whether HbA1c was related to clinical outcomes in diabetic patients undergoing CABG surgery.

**Materials and Methods:**

A literature search was carried out satisfying the predefined inclusion criteria from Pubmed, Embase, and Cochrane Library. Differences were expressed as odds ratios (ORs) with 95% confidence intervals (CIs) to assess the relationships of preoperative HbA1c levels and clinical prognosis in diabetic patients.

**Results:**

7895 diabetic patients undergoing CABG surgery from eight published studies were finally involved in this meta-analysis. Combined analyses revealed that the higher HbA1c level was significantly associated with increased risks of all-cause mortality (OR 1.56, 95%CI 1.29–1.88), myocardial infarction (OR 2.37, 95%CI 1.21–4.64), and stroke (OR 2.07, 95%CI 1.29–3.32) after CABG surgery. However, there was no significant relationship between HbA1c levels and renal failure (OR 2.08, 95%CI 0.96–4.54) in diabetic patients undergoing CABG surgery.

**Conclusions:**

Our meta-analysis demonstrated that the HbA1c level is potentially associated with increased risks of all-cause mortality, myocardial infarction, and stroke in diabetic subjects undergoing CABG surgery. However, further clinical studies with larger sample sizes and longer follow-up period are urgently warranted.

## 1. Introduction

There has been a marked decline in mortality from cardiovascular disease (CVD) over the past several decades [1]. Despite this, as the most common complication among patients with diabetes mellitus (DM), the prevalence of CVD still remains very high. Diabetes mellitus is associated with a two to fourfold higher risk of CVD, as well as an increased risk of mortality by up to threefold [2]. Currently, patients with diabetes mellitus represent about 25% of patients undergoing coronary revascularization [3]. Coronary artery bypass grafting (CABG) surgery is considered a standard of care for patients with coronary artery disease [4]. Epidemiological studies have reported that clinical outcomes after CABG surgery are significantly worse in diabetic patients than in nondiabetic patients [5, 6]. DM increases short-term mortality and morbidity in patients following CABG surgery. The largest study to date by Carson and colleagues examined outcomes in 41,663 diabetic patients compared with those in 105,123 nondiabetic patients and found that patients with diabetes had a 23% to 37% increase in 30-day mortality and in-hospital morbidity compared with patients without diabetes undergoing CABG surgery [7]. Moreover, DM patients undergoing CABG surgery are more likely to develop postoperative infection and as well as new-onset atrial fibrillation and have worse clinical outcomes than non-DM patients [8].

It has been observed that glycemic control is associated with increased short- and long-term mortality in diabetic patients undergoing CABG surgery. 2011 ACCF/AHA Guideline for Coronary Artery Bypass Graft Surgery stated that the use of continuous intravenous insulin to achieve and maintain an early postoperative blood glucose concentration ≤ 180 mg/dL while avoiding hypoglycemia is indicated to reduce the incidence of adverse events, including deep sternal wound infection, after CABG [9]. Several studies show that acute myocardial infarction (AMI) with hyperglycemia at admission had higher adverse cardiac events than that with normal blood glucose, and most studies found that hyperglycemia admission, such as fasting, postprandial, or incidental glycemia, is associated with increased short- and long-term mortality in diabetic patients after CABG [10–12]. However, AMI with acute stimuli at admission will lead to hyperglycemia with higher secretion of catecholamine, which is very uncontrollable and unstable [13]. Thus, the relatively long-term glucose metabolic state, which is assessed by glycosylated hemoglobin (HbA1c), rather than a snapshot of blood glucose at a single time point, has been transferred to predict the clinical prognosis in diabetic patients with AMI. HbA1c, an established indicator of relatively long-term blood glucose control, can reflect the average blood glucose levels during the previous 2 to 3 months. It is potentially a better prognostic predictor than other glucose metabolic parameters, which only exclusively reflect incidental, fasting, or postprandial blood glucose in diabetic patients [14, 15].

Several studies have evaluated the potential effects of HbA1c levels on clinical implications in diabetic patients undergoing CABG surgery. However, these studies were contradictory and inconclusive, due to small sample size in most cohorts. The lack of adequate power is insufficient to elucidate the association between HbA1c levels and clinical outcomes. Meta-analysis is a very powerful approach to synthesize data from varied studies on the same issue. Therefore, the aim of this study was to analyze the association between HbA1c levels and clinical outcomes in diabetic patients who were undergoing CABG surgery. To the best of our knowledge, this is the first meta-analysis to evaluate the relationships between the quality of preoperative glycemic control, as assessed by plasma HbA1c levels, and the progression of clinical prognosis in diabetic patients who were undergoing CABG surgery.

## 2. Methods

This meta-analysis was conducted based on Preferred Reporting Items for Systematic Reviews and Meta-Analysis (PRISMA) (Supplementary Table 1 available online at https://doi.org/10.1155/2017/1537213). The literature search, data extraction, and quality assessment were undertaken independently and blindly by two authors (JZ and JC) using a standardized approach. Any disagreements were resolved by a third reviewer (XHX).

### 2.1. Data Sources, Search Strategy, and Selection Criteria

The databases of Pubmed, Embase, and Cochrane Library databases were comprehensively searched for relevant studies. The main search term was a combination of MESH terms and text words for DM, HbA1c, and CABG, with the following terms: “HbA1c” OR “glycosylated hemoglobin A1c” OR “glycemic control” AND “coronary artery bypass graft” OR “CABG” AND “diabetes mellitus”. All literatures were published up to October 2016 and the language was limited to English. Additional relevant references quoted in searched articles were also selected. Endnote X7 performed all literature management.

Studies that examined HbA1c levels and clinical prognosis in diabetic patients undergoing CABG surgery were included. Studies with the following criteria were included: (1) measured HbA1C levels; (2) case-control studies on the relationship between HbA1C levels and clinical outcomes; (3) sufficient data for evaluating odds ratios (ORs) with 95% confidence intervals (95% CIs). Studies were excluded if they satisfied the following criteria: (1) studies in which HbA1C levels could not be ascertained; (2) reviews or abstracts; (3) animal studies. For the overlapping studies, only the one with the largest sample size was included in our meta-analysis.

### 2.2. Data Extraction

Data was extracted from each selected study using a standardized protocol, with a predesigned review form: author, publication year, country, study design, total numbers of cases and controls (sample size), demographics, follow-up period and rates, and clinical outcomes. Absolute numbers were recalculated when percentages were reported. Authors of the identified studies were contacted via e-mail if further study details were needed. Three reviewers discussed and decided on the final inclusion of studies for this review and meta-analysis (JZ, JC, and XHX).

### 2.3. Assessment of Study Quality

The Newcastle–Ottawa scale (NOS) was utilized to systematically evaluate the study quality (http://www.ohri.ca/programs/clinical_epidemiology/oxford.asp). Specifically, all the studies were judged based on these following three elements, including the selection of the study groups (0–4 points), the ascertainment of either the exposure or outcome of interest (0–3 points), and the comparability of the groups (0–2 points), which was reported as our previous study [16].

### 2.4. Statistical Analysis

RevMan 5.3 software, developed by the Cochrane Collaboration (http://tech.cochrane.org/revman/, accessed on June 13, 2014.), was used for this meta-analysis. Pooled ORs were reported with 95% CIs, and a two-tailed *p* < 0.05 was considered statistically significant for all analyses. The Cochran's *Q* test and *I*^2^ test were all performed to judge the heterogeneity among the studies included in this meta-analysis. Heterogeneity was also considered to be significant at *p* < 0.1 for the *Q* statistic. *I*^2^ values of 25%, 50%, and 75% corresponded to low, moderate, and high levels of heterogeneity, respectively [17]. Applying the fixed-effects model or random-effects model depended on the degree of heterogeneity among studies. Results showing no significant heterogeneity were analyzed by the fixed-effects model and those with significant heterogeneity were analyzed by the random-effects model [18]. Sensitivity analysis was carried out by successively excluding the low-quality studies to assess the stability of the outcomes [19]. Potential publication bias was assessed by visual inspection of the funnel plot, and an asymmetric plot suggested possible publication bias [20].

## 3. Results

### 3.1. Studies Included and Participant Characteristics

**Figure 1:**
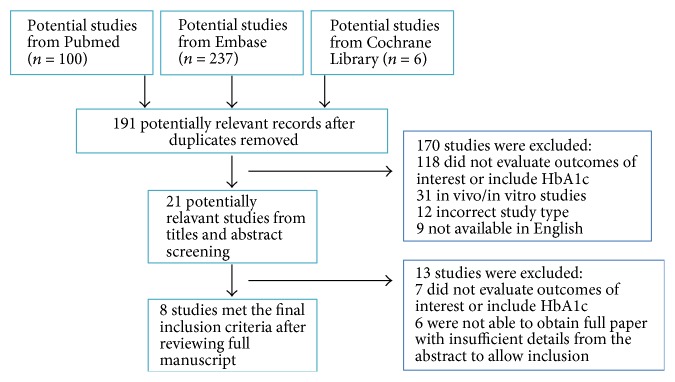
Study flow chart of study selection and exclusion.

Figure 1 summarizes the selection of reports of eligible clinical studies. We identified 343 potentially eligible literature citations and 191 were kept after removing duplicates. 21 potential studies were further reviewed after reading the title and abstract. Finally, only 8 reports of studies with suitable data were included in the final meta-analysis [21–28]. A total of 7895 subjects were enrolled in the studies. The detailed characteristics of the studies included in the meta-analysis are given in Table 1. These studies were performed in seven countries (Canada, the United States, Poland, Sweden, Argentina, Iran, and Japan). Four studies were aimed to evaluate the in-hospital outcomes [21, 23, 25, 28] and other studies were to assess the long-term outcomes discharged from the hospital [22, 24, 26, 27]. The enrollment sample size ranged from 96 to 3201 subjects. In accordance with the American Diabetes Association guidelines [29], diabetic patients were stratified based on preoperative glycemic control. Of this eight studies, six studies indicated that “optimal glycemic control” was defined as HbA1c ≤ 7% and “suboptimal glycemic control” was defined as HbA1c > 7%. The remaining two studies showed that the cutoff point of HbA1c was 6.9% and 6.5%. The eight studies were mostly with a NOS score of ≥7. Therefore, they improved the quality of the final results [30].

### 3.2. HbA1c Levels and Clinical Outcomes

#### 3.2.1. HbA1c Levels and All-Cause Mortality

**Figure 2:**
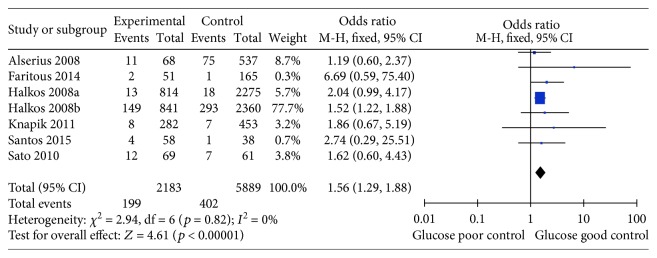
Forest plot of the relationship between HbA1c level and all-cause mortality.

Seven studies assessed the relationship between HbA1c levels and all-cause mortality in diabetic patients undergoing CABG surgery [21–27]. Comprehensive integration and analyses revealed a significant correlation between higher HbA1c levels and increased risks of all-cause mortality (OR 1.56, 95%CI 1.29–1.88, *p* < 0.001), with very low heterogeneity (*I*^2^ = 0%; *p* = 0.82; Figure 2).

#### 3.2.2. HbA1c Levels and Myocardial Infarction

**Figure 3:**
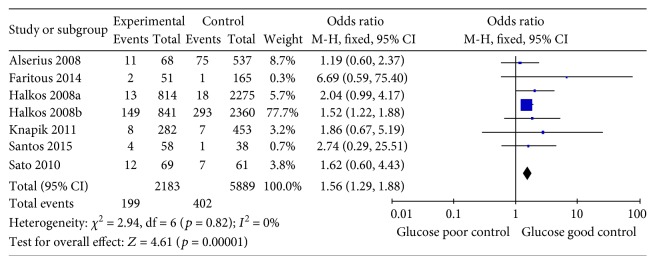
Forest plot of the relationship between HbA1c level and myocardial infarction.

Myocardial infarction is characterized by ischemia-induced percutaneous or surgical revascularization of the treated vessel. Five studies were included to evaluate the relationship between HbA1c levels and the development of myocardial infarction among diabetic patients undergoing CABG surgery [22, 24, 26–28]. Combined analyses revealed a significant correlation between higher HbA1c levels and the risk of myocardial infarction (OR 2.37, 95%CI 1.21–4.64, *p* = 0.01), with low heterogeneity (*I*^2^ = 0%; *p* = 0.73; Figure 3).

#### 3.2.3. HbA1c Levels and Stroke

**Figure 4:**
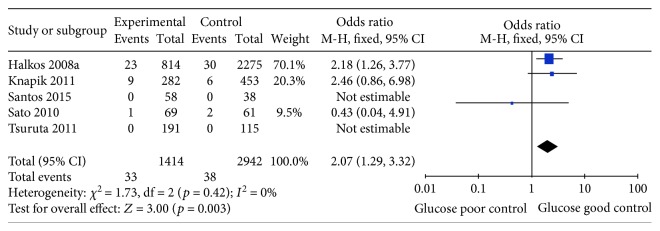
Forest plot of the relationship between HbA1c level and stroke.

Stroke is a severe complication following CABG surgery, which was defined as an acute neurologic deficit of presumed vascular origin lasting more than 24 hours, or the presence of brain infarction on neuroimaging. Five studies were included to assess the effect of HbA1c levels and stroke among diabetic patients undergoing CABG surgery [21, 22, 24, 26, 28]. The analysis indicated that HbA1c levels were positively correlated with the risk of stroke after CABG surgery (OR 2.07, 95%CI 1.29–3.32, *p* = 0.003). The heterogeneity was also very low (*I*^2^ = 0%; *p* = 0.42; Figure 4).

#### 3.2.4. HbA1c Levels and Renal Failure

**Figure 5:**
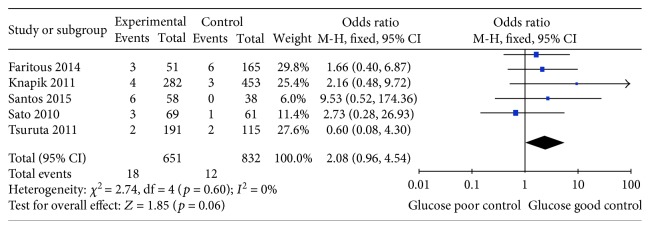
Forest plot of the relationship between HbA1c level and renal failure.

Previous studies have demonstrated that chronic kidney disease is an independent risk factor for postoperative events following CABG surgery and renal failure has been reported as associated with increased risk of morbidity and mortality after CABG surgery [31]. Five studies assessed the effect of HbA1c levels and renal failure among diabetic patients undergoing CABG surgery [21, 24, 26–28]. No significant association was found between higher HbA1c levels and renal failure (OR 2.08, 95% CI 0.96–4.54, *p* = 0.06), with low heterogeneity (*I*^2^ = 0%; *p* = 0.60; Figure 5).

### 3.3. Sensitivity Analysis and Publication Bias

**Figure 6:**
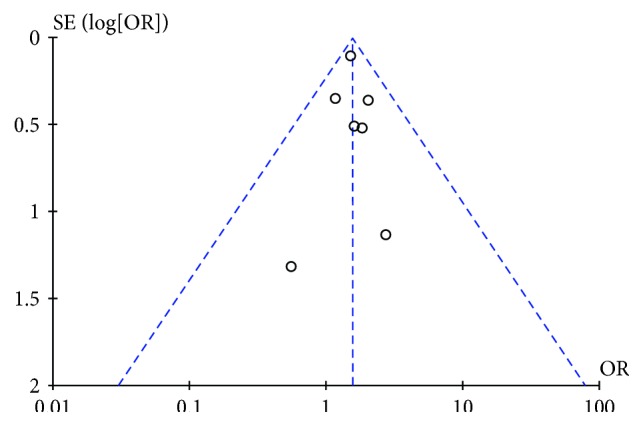
Funnel plot of publication bias in terms of HbA1c level and all-cause mortality in all diabetic patients.

To examine the stability of the pooled results, a sensitivity analysis was performed by the one-at-a-time method, with consecutively excluding one study at a time and repeating the meta-analysis. If the omission of one study significantly changed the result, it implied that the result was sensitive to the studies included. Our study showed that the corresponding summary ORs were not changed significantly, indicating a statistically robust result (data not shown). Potential publication bias was assessed by visual inspection of the funnel plot, and an asymmetric plot suggested possible publication bias. Funnel plots' shape of all studies showed symmetry and revealed no publication bias in all studies included in the meta-analysis studies in terms of all-cause mortality (Figure 6).

## 4. Discussion

Diabetes mellitus has long been recognized as an independent risk factor for the development of coronary artery disease [32], and it is associated with a 2- to 4-fold increased risk of cardiovascular disease, with event rates correlating with the degree of hyperglycemia [33]. HbA1c values have been widely investigated as an index of long-term blood glucose control and outcome predictors in diabetic patients. In a large multiethnic cohort, an increase of 1% in HbA1c was associated with an increased risk of 18% in cardiovascular disease events [34], 19% in myocardial infarction [34], and 12% to 14% in all-cause mortality [4, 35]. A meta-analysis of data from 33,040 participants in five prospective randomized controlled trials reported that intensive glycemic control with a 0.9% decline in HbA1c concentration resulted in a 17% reduction in events of nonfatal myocardial infarction and a 15% reduction in events of coronary heart disease in patients with DM [36]. In 2015, an undated scientific statement from the American Heart Association and the American Diabetes Association has been recommended for the benefit of glycemic control on cardiovascular disease in diabetic patients [37]. However, it is unknown whether adequacy of diabetic control, measured by HbA1c, is a reliable predictor of adverse outcomes after CABG surgery. Several studies are about the prognostic role of HbA1c levels in diabetic patients following CABG surgery. However, the results remained conflicting and unclear. Therefore, the optimal HbA1C level in diabetic patients is a subject of ongoing controversy that may be especially pertinent in diabetic patients with CABG surgery.

In this meta-analysis, eight case-control studies about HbA1c levels and the clinical outcomes in diabetic patients after CABG surgery were analyzed. It revealed a significant correlation between higher HbA1c levels and the risk of all-cause mortality (OR 1.56, 95%CI 1.29–1.88), myocardial infarction (OR 2.37, 95% CI 1.21–4.64), and stroke (OR 2.07, 95%CI 1.29–3.32) after CABG surgery. However, the exact mechanism underlying the association between higher HbA1c levels and these poor clinical outcomes following CABG surgery has not been fully elucidated yet. Several possible mechanisms may explain the association. First, increased HbA1C could be a signal of previous poor glycemic control and “metabolic memory” suggests that diabetic cardiovascular disease can persistently exist even after glucose normalization in diabetic patients [38]. Second, higher HbA1C concentrations were commonly associated with metabolic syndrome, such as obesity, hypertension, and dyslipidemia, which can increase the risks of poor clinical outcomes. Third, chronic hyperglycemia can cause vascular endothelial cell damage with increased cellular proliferation [39], which can lead to myocardial infarction and stroke after CABG surgery. However, no significant association was observed between HbA1C levels and renal failure. This may be because the definition of renal failure is variable among studies and further studies should specify the definition of renal failure. These data suggest that higher HbA1c concentrations may have potential clinical implications in diabetic patients undergoing CABG surgery.

However, several limitations should be taken into consideration in interpreting our results. First, the total numbers of cases and controls in this meta-analysis were limited, which may be insufficient to demonstrate the association between HbA1C levels and clinical outcomes and the smallest study only enrolled 96 patients [26]. Second, several cardiac parameters, such as such as ejection fraction, coronary atherosclerosis, number of bypasses, and comorbidities, which are potential confounders and were not included in several studies, may limit the effect size of our results. Thus, these cardiac parameters should be included and adjusted in further studies. Third, the different results between hard endpoints (death and AMI) might be attributed to the relatively short follow-up durations. The follow-ups were relatively short, and that was about three years in four studies and less than one month in other studies. Fourth, the languages of included studies were limited to English and it may cause publication bias, due to the absence of some studies in some other languages. Some other limitations are inherent to the available literature, including the observational nature of studies, the type of diabetes mellitus, and unclear follow-up rates in several studies. Thus, more large-scale, multinational, multicenter, randomized, controlled, and long-term follow up trials are warranted.

To the best of our knowledge, our present study is the first meta-analysis to assess the association between HbA1c levels and the progression of clinical outcomes in diabetic patients who were undergoing CABG surgery. Although there are some aforementioned limitations, this systematic analysis was statistically more persuading than any single study. It reached a strong conclusion that higher HbA1c levels (>7%) may be a potential risk factor of all-cause mortality, myocardial infarction, and stroke in diabetic patients undergoing CABG surgery. In conclusion, our results is novel in showing that preoperative HbA1c levels, a parameter of long-term glycemic control, not a snapshot of blood glucose at a single time point, play an important role in the prognosis of diabetic patients undergoing CABG. Meanwhile, in order to better assess the association between HbA1c levels with the clinical outcome among diabetic patients following CABG surgery, further clinical studies with larger sample sizes should be required to verify the association and further studies to clarify the underlying mechanisms are urgently warranted.

## Supplementary Material

The Preferred Reporting Items for Systematic Reviews and Meta-Analysis (PRISMA) in this meta-analysis.

## Figures and Tables

**Table 1:** Characteristics of the studies included in the meta-analysis.

Study ID	Year	Country	Study design	Sample size	Male (%)	Follow-up duration	Follow-up rate (%)	HbA1c levels	Study outcomes	NOS score
Sato et al. [21]	2010	Canada	Prospective cohort	130	70.8	30 days	100%	<6.5% versus ≥6.5%	All-cause mortality, myocardial failure, stroke, dialysis, serious infection	4/3/2
Halkos et al. [22]	2008	USA	Prospective cohort	3089	72.6	In-hospital stay	100%	≤7% versus >7%	All-cause mortality, MI, renal failure, atrial fibrillation, cerebrovascular accident	4/2/1
Halkos et al. [23]	2008	USA	Prospective cohort	3201	71.6	2.8 ± 1.4 years	NA	≤7% versus >7%	Cerebrovascular disease, renal insufficiency, CHF, MI, peripheral vascular disease	3/2/1
Knapik et al. [24]	2011	Poland	Prospective cohort	735	66.3	In-hospital stay	100%	≤7% versus >7%	All-cause mortality, stroke, renal failure, wound infection, perioperative MI	4/3/2
Alserius et al. [25]	2008	Sweden	Prospective cohort	122	54.9	3.5 years	100%	≤7% versus >7%	All-cause mortality, superficial sternal wound infections, mediastinitis	4/1/2
Santos et al. [26]	2015	Argentina	Prospective cohort	96	82.3	In-hospital stay	NA	≤7% versus >7%	All-cause mortality, CHF, MI, renal failure, stroke	4/2/2
Faritous et al. [27]	2014	Iran	Prospective cohort	216	63.9	In-hospital stay	NA	≤7% versus >7%	All-cause mortality, arrhythmia, AMI, CHF	4/1/2
Tsuruta et al. [28]	2011	Japan	Prospective cohort	306	79.1	3.6 ± 1.7 years	100%	<6.5% versus ≥6.5%	MI, arrhythmia, congestive heart failure, sudden death	4/3/2

CHF: congestive heart failure; MI: myocardial infarction; NA: not available.
